# Multi-scale instrumental analyses of plasticized polyhydroxyalkanoates (PHA) blended with polycaprolactone (PCL) and the effects of crosslinkers and graft copolymers†

**DOI:** 10.1039/c8ra10045d

**Published:** 2019-01-11

**Authors:** Masakazu Nishida, Tomoko Tanaka, Yoshio Hayakawa, Takashi Ogura, Yoshiaki Ito, Masahiro Nishida

**Affiliations:** National Institute of Advanced Industrial Science and Technology (AIST) 2266-98 Shimoshidami, Moriyama-ku Nagoya 463-8560 Japan m-nishida@aist.go.jp +81 52 736 7406 +81 52 736 7493; Nagoya Institute of Technology Gokiso-cho, Showa-ku Nagoya Aichi 466-8555 Japan

## Abstract

Details of the mechanism underlying the tensile properties of plasticized polyhydroxyalkanoates (PHA) including poly(butylene succinate) (PBS) were investigated by blending with poly(ε-caprolactone) (PCL) as well as the addition of compatibilizers. Multi-scale instrumental analyses employed micro-focus X-ray CT to provide micro-scale morphology information on the order of ten microns while solid-state NMR spectral and relaxation time analyses contributed knowledge of the environment and molecular mobility of each constituent at the molecular to nano-scale. The blend of plasticized PHA with 50% PCL adopted a sea-island morphology to improve elongation at break in a quasi-static tensile test, which was dominated by the tensile properties of the added PCL. However, impact tensile properties were less improved by PCL addition, because its molecular mobility was suppressed by blending. Meanwhile, peroxy crosslinkers changed the sea-island morphology to homogenous in X-ray CT observations. Although the homogenous morphology sharply lowered the elongation at break in a quasi-static tensile test, the homogenous morphology improved impact tensile properties. Furthermore, graft polymers having acrylonitrile–styrene side-chains did not change the sea-island morphology but increased the molecular mobility of PBS in the plasticized PHA. This weak interaction between the plasticized PHA and PCL improved tensile properties in both quasi-static and impact tensile tests.

## Abstract 2

Multi-scale instrumental analyses showed that the crosslinker changed the morphology to homogenous while the graft polymer increased molecular mobility.

## Graphical abstract

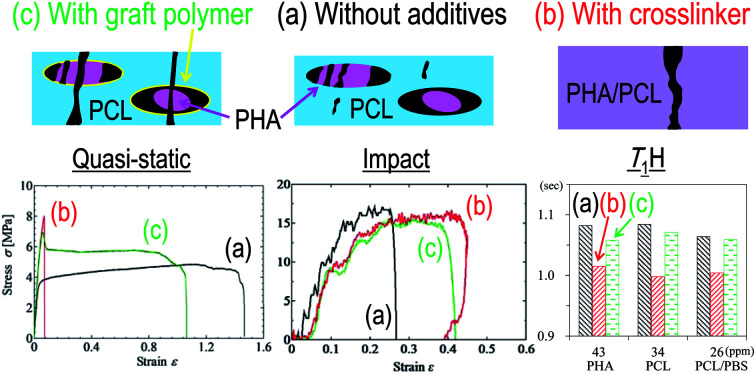

## Introduction

As part of the solution to global climate problems for a sustainable society, it is desirable to replace petroleum-based polymers with biodegradable equivalents. Polyhydroxyalkanoates (PHA), which are made by a microbial process, have the advantage of consuming non-edible materials as the feedstocks.^1^ PHA is a family of biopolyesters commonly accumulated by many bacteria, consisting of the following polyesters: poly-3-hydroxybutylate (PHB), poly-3-hydroxyvalerate (PHV), as well as their isomeric polymers and copolymers. The control of chemical structure of the PHA homopolymers and copolymers in both microbial and chemical production is still under development.^2^ Applications of PHA have extended to various industrial fields with developments in characterization and production. In particular, PHA is useful in medical sectors, such as tissue engineering, bio-implants, drug delivery, and surgical materials, as well as in nanotechnology.^3^ Other industrial applications include packaging, moulded goods, paper coatings, non-woven fabrics, adhesives, films, and performance additives.^4^ Considering these applications, the chemomechanical properties of PHA, which are influenced by polymer composition and microstructure, have been investigated.^5^

Chemical modification approaches can alter the characteristics of PHA, such as its mechanical properties, surface structure, amphiphilic character, and rate of degradation.^6^ Hydroxylation, carboxylation, epoxidation, and chlorination in particular have been considered for use in biomedical applications.^7^ Blending of PHA with other polymers is the most important method for controlling its thermal and crystallization behaviour and morphology which are closely related to mechanical properties and biodegradability.^8^ The morphology of poly(3-hydroxybutyrate-*co*-3-hydroxyvalerate) (PHBV)/polylactide (PLA) blends was studied to assess the correlation to thermal, rheological and barrier properties.^9^ Depending on the application, bio-based PLA/PHB blend can be manufactured in various shapes. Plasticized PLA/PHB blends with an oligomer of lactic acid, which was extruded for the film formation process, showed a decrease in the glass transition temperature while maintaining the mechanical properties.^10^ The plasticized PLA/PHB blend could be also produced as flexible fibres by the electrospinning method and the blend ratio affected the biodegradability of the electrospun fibres in composting conditions due to their crystallinities.^11^ In order to improve melt processability and thermomechanical properties, PHA was blended with poly(butylene adipate-*co*-terephthalate) (PBAT) by melt extrusion to demonstrate that an acid wash dramatically improved processability.^12^ Binary blends for PHB and poly(ε-caprolactone) (PCL) blend that were manufactured by a twin screw co-rotating extruder and were subsequently injection moulded showed impact that depended on the PCL content.^13^

Since PHA is brittle and hydrophilic, it has disadvantages for processability and a small amount of compatibilizer has sometimes been used in blending PHA; the morphology and mechanical properties of the PHA-based blend were changed by compatibilizers, including crosslinkers and graft copolymers. The reactive polymer blending technique changed the physicochemical interactions between the constituent polymers because of *in situ* formation of the compatibilizing agents, such as a branching/crosslinking copolymer and a graft copolymer.^14^ An epoxy compatibilizer reacted with the OH group of poly(3-hydroxybutyrate-*co*-3-hydroxyhexanoate) (PHBH) and the OH/COOH groups of PLA to improve the elongation at break and impact strength of the PHBH/PLA blend.^15^ Dicumyl peroxide, which acted as a free-radical grafting initiator, compatibilized PHBV/PBS and PHA/PBS blends to ameliorate tensile strength, impact toughness, and elongation at break of injection-moulded blends.^16^ Grafting maleic anhydride improved the miscibility of a PHBV/PBS blend with incorporation of sepiolite because of a synergistic effect induced by both compatibilizer and filler.^17^ Starch-based materials can be used as compatibilizer for melt-blending with PHA and PBAT, inhibiting the secondary crystallization of the PHA component in the cast films. Such approaches show promise for flexible packing materials.^18^

As shown above, the blending of PHA as well as the compatibilizing of PHA-based blends obviously improved the material characteristics of PHA; however, in order to know the details of the mechanism of their functional expression, an analytical method that covers molecular- to nano-scale length scales is necessary. Solid-state NMR is a useful analytical method in particular for both synthetic and natural polymers over these small-scale orders; it has provided significant information about molecular structures and dynamics for amorphous and crystalline phases of PHB^19^ as well as PHB-based copolymers and blends.^20,21^ Despite the correlation of microscopic properties to not only the morphology, but also to the mechanical properties of PHA and related materials, solid-state NMR has not yet been applied to analyses of structures and dynamics from the molecular- to nano-scale orders. Using the multi-scale instrumental analyses that range from molecular- to nano-scale (solid-state NMR) to micro-scale (morphology by SEM observation), we have evaluated the material characteristics of biomass polymers, for example, changes of biomass constituents of soft wood^22^ and other plant materials.^23^

The present study aims to extend multi-scale instrumental analyses to the multicomponent PHA system to reveal changes at the molecular- to micro-scales that contribute to their mechanical properties. Our preceding paper has revealed the influence of molecular mobility on the compatibility of PHA and PBS for plasticized PHA (commercially available PHA4422P), using solid-state NMR spectral and relaxation time analyses not only at ambient and variable temperatures.^24^ In a quasi-static tensile test of this plasticized PHA blending with PCL, the addition of 50% PCL dramatically increased the elongation at break and the specimens became pours shape.^25^ In a dynamic tensile test using a split Hopkinson bar (SHPB) method, a blend consisting of 50% plasticized PHA and 50% PCL gave small elongation at break, which increased with the addition of compatibilizers.^26^ Here, we focus on plasticized PHA (PHA4422P) blended with various amounts of PCL and with various compatibilizers (crosslinkers and graft polymers). By combining multi-scale instrumental analyses including solid-state NMR spectral and relaxation time analyses and micro-focus X-ray computed tomography (CT) with newly collected systematic tensile test data, the details of the mechanism behind their mechanical properties have been elucidated.

## Experimental

### Materials

The plasticized PHA pellet, PHA4422P, was purchased from G5 Manufacturing, Singapore. The PHA4422P pellet consisted of 65% PHA, 30% PBS, and 5% crosslinking reagent. The PCL pellet, Capa 6800, was purchased from Perstorp Japan Co. Ltd., Tokyo, Japan. The crosslinking reagents, PERHEXA 25B (liquid, over 90% purity) and PERHEXA 25B-40 (mixed with silica as a white solid, 40% purity), were purchased from NOF CORPORATION, Tokyo, Japan. The graft polymers, Modiper A4400 and Modiper CL430-G, were purchased from NOF CORPORATION, Tokyo, Japan. Modiper A4400 was an ethylene–glycidyl methacrylate copolymer with acrylonitrile–styrene copolymer. Modiper CL430L was polycarbonate with a functional group-modified acrylonitrile–styrene copolymer. All materials were used without further purification. Raw pellets and compatibilizers (1% crosslinkers and 1% graft polymers) were melt-mixed at 175 °C for 20 min at a rotor speed of 50 rpm with a conventional melt-mixer. The mixed sample was moulded into plates of 5 mm thicknesses at 175 °C and 30 MPa for 30 min with a hot press.

### Static tensile test

Static tensile test specimens with a gauge area of 5 mm × 2 mm and a gauge length of 10 mm were made from the plates using machine processing. Static tensile tests were carried out at a crosshead speed of 0.2 mm min^−1^ using a universal testing machine. Nominal strain was obtained by images of the gauge length, taken by a digital camera in the elastic region and by the displacement of the crosshead in the plastic region. Nominal stress was calculated using the output of load cell at the crosshead.

### Dynamic tensile test

Dynamic tensile test specimens with a gauge area of 2 mm × 5 mm and a gauge length of 4 mm were made from the plates using machine processing. At high strain rates, the dynamic properties of the specimens were examined by the tensile split Hopkinson bar test.^27^ The diameters and lengths of input and output bars were 12 mm and 2000 mm respectively. Strain gauges were applied to both sides of the input and output bars at distances of 1750 mm and 350 mm from the specimen, respectively.

### Micro-focus X-ray CT observations

Micro-focus X-ray images were recorded with a FLEX-M345CT (Beamsense Co. Ltd., Osaka, Japan) instrument at an acceleration voltage of 40 kV and a tube current of 100 μA. The sample was rotated over 180° at 0.5° steps and two transmission images (the pixel size is 2.12 μm × 2.12 μm to 3.07 μm × 3.07 μm) were acquired using a charge-coupled device (CCD) with the exposure time of 1500 ms. The CT images were obtained by slicing the 3D image reconstructed from the transmission images.

### Solid-state NMR spectrum measurements

Magic angle spinning (MAS) nuclear magnetic resonance (NMR) spectra were measured on a Varian 400 NMR system spectrometer (Palo Alto, CA) with a Varian 4 mm double-resonance T3 solid probe. The samples were placed in a 4 mm ZrO_2_ rotor spun at 15 kHz over a temperature range of 20–22 °C. ^1^H MAS NMR spectra were measured at 399.86 MHz for the ^1^H nuclei and were collected with a 40 ms acquisition period over a 30.5 kHz spectral width in 16 transients, and a 3 s recycle delay. ^13^C MAS NMR spectra were collected with a 2.6 μs π/2 pulse at 100.56 MHz for the ^13^C nuclei and a 40 ms acquisition period over a 30.7 kHz spectral width. Proton decoupling was performed with an 86 kHz ^1^H decoupling radio frequency with a small phase incremental alteration (SPINAL) decoupling pulse sequence. Cross-polarization/magic angle spinning (CP-MAS) NMR spectra were measured with a 5.0 s recycle and 1024 transients delay, using a ramped-amplitude pulse sequence with a 2 ms contact time and a 2.5 μs π/2 pulse for the ^1^H nuclei. The amplitude of the ^1^H pulse was ramped up linearly from 90.5% of its final value during the cross polarization contact time. Pulse saturation transfer/magic angle spinning (PST-MAS) NMR was measured used the single π/2 pulse for the ^13^C nuclei with a 5 s recycle delay in 2048 transients after saturation of ^1^H nuclei with 13 consecutive 2.5 μs pulses and a 27.5 μs delay.

### Nuclei magnetic relaxation analyses

The ^1^H spin-lattice relaxation time in the laboratory frame (*T*_1_H) was indirectly measured *via* detection of ^13^C resonance enhanced by cross-polarization, applied after a π pulse to ^1^H nuclei with the inversion recovery method. The ^13^C spin-lattice relaxation time in the laboratory frame (*T*_1_C) was measured with the conventional Torchia's pulse sequence.^28^ The *T*_1_ analyses were performed with the same solid-state probe with the same contact time and acquisition period used for the ^13^C CP-MAS NMR spectrum. The ^1^H spin-lattice relaxation in the rotation frame (*T*_1ρ_H relaxation) was examined by the observation of signal decay curve with variable contact time over a range of 100–12 000 μs in CP-MAS NMR sequence.

## Results and discussion

### Effects of PCL ratio and compatibilizers on tensile properties of plasticized PHA/PCL blends

**Fig. 1:**
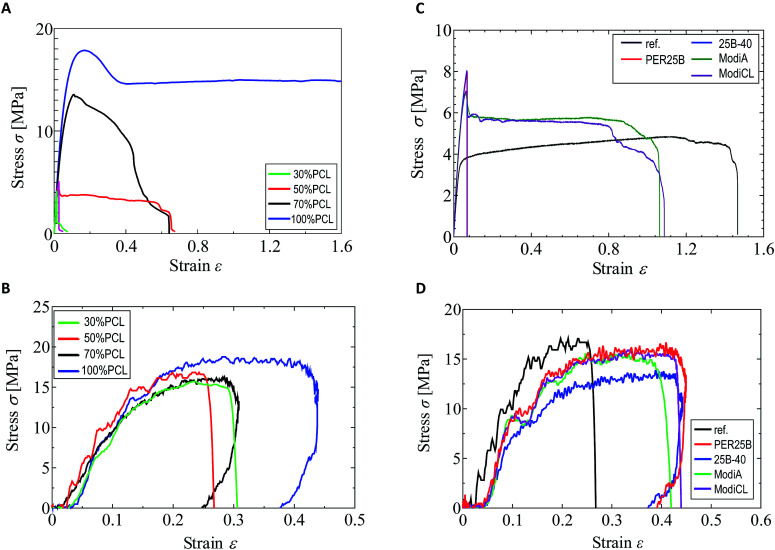
Quasi-static tensile test and impact tensile test of plasticized PHA/PCL blends. (A) Quasi-static (plasticized PHA/PCL blend). (B) Impact (plasticized PHA/PCL blend). (C) Quasi-static (50% PCL with compatibilizers). (D) Impact (50% PCL with compatibilizers).

As shown in our previous paper,^25^ the tensile strength and elongation at break in a quasi-static tensile test were significantly changed by addition of PCL to the plasticized PHA, PHA4422 (PHA/PBS copolymer), depending on the PCL ratio. In particular, over 50% PCL addition increased both the strength and elongation in the quasi-static test. In contrast, the value of the PCL ratio scarcely influenced the impact tensile test of the plasticized PHA/PCL blend. The different effects of the PCL ratio and compatibilizers between the quasi-static and impact tensile tests can be observed in Fig. 1. Comparing the quasi-static (Fig. 1A) and impact (Fig. 1B) tests, the 100% PCL specimen has a smaller elongation at break in the impact test than that in the quasi-static test. The plasticized PHA/PCL blend showed less difference with the PCL content in the impact test than in the quasi-static test, owing to the above tensile properties of PCL.

The tensile strength and elongation at break of the 50% plasticized PHA/50% PCL blend in the impact test noticeably increased with only 1% compatibilizers, such as the crosslinkers (PERHEXA 25B, PERHEXA 25B-40) and graft polymers (Modiper A4400, Modiper CL430L), as previously communicated.^26^ In the preliminary experiments, the addition amount of the compatibilizer was not greatly different between 1% and 5%. Thus, the addition amount of the compatibilizer was examined for 1% in this study. The additives produced different effects in the quasi-static (Fig. 1C) and impact (Fig. 1D) tests, as well as with changes of the PCL ratio. In the quasi-static test, the crosslinkers significantly decreased elongation at break while they increased tensile strength. The graft polymers in the quasi-static test also increased tensile strength, but they only slightly decreased elongation at break compared with the original blend.

### Morphological observations of plasticized PHA/PLA blends with micro-focus X-ray CT

**Fig. 2:**
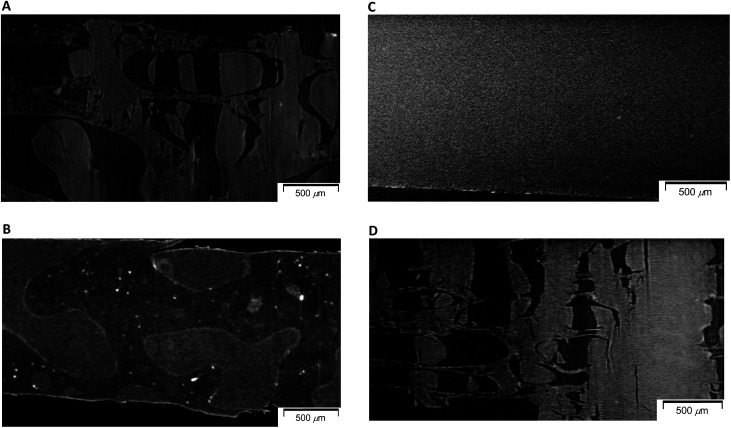
Micro-focus X-ray CT images of 50% plasticized PHA/50% PCL blends. (A) Without compatibilizers after quasi-static test. (B) Without compatibilizers after impact test. (C) With PERHEXA25B after quasi-static test. (D) With Modiper A4400 after quasi-static test.

In order to examine changes of 50% plasticized PHA (PHA/PCL copolymer)/50% PCL blend due to the compatibilizers in terms of micro-scale order, morphological changes of specimens after quasi-static and impact tensile tests were observed using soft-focus X-ray CT for specimens having fractures of different shapes. The morphological difference of 50% plasticized PHA/50% PCL blend after the quasi-static and impact tests is shown in Fig. 2. One can see that a sea-island structure having relatively large island domains (<1000 μm) was observed in both specimens. In the specimen after quasi-static test (Fig. 2A), the island portion was broken to several sections while the sea portion was stretched with minute voids. This morphology after the quasi-static test corresponded to the porous structure previously observed in the visible and low magnification X-ray CT images.^25^ In contrast, after the impact test (Fig. 2B), both island and sea shapes were not deformed and showed almost no stretching. Since the 100% PCL specimen has a large elongation at break in the quasi-static test while it had a small elongation in the impact test, the sea portion was assigned as PCL and the island portion was assigned as PHA.

Since the effect of compatibilizers appeared in the quasi-static tensile test more clearly, morphological changes of the 50% plasticized PHA/50% PCL blend due to the compatibilizers were examined for the specimens after the quasi-static test using micro-focus X-ray CT. The crosslinker (1% PERHEXA 25B) changed the sea-island structure of plasticized PHA/PCL blend into a homogenous morphology over tens of micron (Fig. 2C). In this case, neither visible cracks nor deformations were observed, being accountable for the very small elongation at break in the quasi-static test. Meanwhile, the graft polymer (1% Modiper A4400) maintained the sea-island structure of the plasticized PHA/PCL blend; however, both the island and sea regions were broken and elongation of sea portion was also observed at the same time (Fig. 2D). Therefore, the graft polymer enhanced the interaction between plasticized PHA and PCL resulting in the fracture of the sea portion consisting of PCL caused by low elongation of PHA.

### ^1^H MAS NMR spectra of plasticized PHA/PLA blends

Since ^1^H nuclei have large dipolar–dipolar interactions with each other, solid-state ^1^H NMR spectra have wide lines even when using magic-angle spinning (MAS), which can offset the anisotropy of dipoles. The line width of ^1^H MAS NMR spectra is affected by the spin–spin relaxation (*T*_2_ relaxation) which is suppressed by molecular motions. For example, water molecules had a narrower line width because of a longer *T*_2_ (greater molecular mobility) while biomass constituents in soft wood showed a wider line shape because of shorter *T*_2_ (less molecular mobility).^29^ Here, the molecular mobility involved in *T*_2_ relaxation could be monitored by ^1^H MAS NMR spectra of plasticized PHA/PCL blends.

**Fig. 3:**
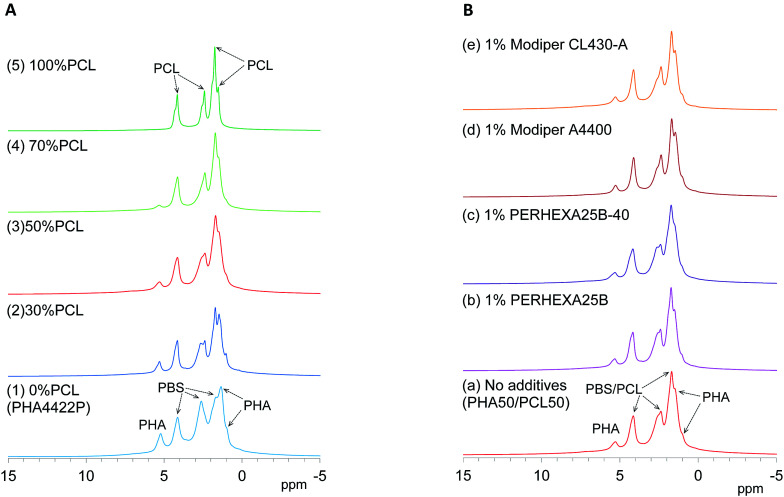
^1^H MAS NMR spectral changes of plasticized PHA/PCL blends. (A) PCL ratio. (B) Compatibilizers.

First, changes of ^1^H MAS NMR spectra of plasticized PHA/PCL blends with PCL ratio are shown in Fig. 3A. Although both pre-plasticized PHA and PBS copolymer had broad low-intensity signals as shown in Fig. S1 (ESI†), the plasticized PHA (PHA4422P) showed relatively sharp signals not only in the PHA region but also in the PBS region [Fig. 3A(1)]. Therefore, the plasticization of PHA with PBS enhanced molecular motions of both PHA and PBS because of the suppression of *T*_2_ relaxation. Like the plasticized PHA, flexible PCL showed sharp high-intensity signals [Fig. 3A(5)]. All plasticized PHA blends including 30%, 50%, and 70% PCL [Fig. 3A(2)–(4)] also showed similar line shapes and intensities to the plasticized PHA and flexible PCL. Thus, molecular mobility concerned with *T*_2_ relaxation was little changed by blending with flexible PCL because the copolymerization with PBS has already amplified the molecular mobility of the constituent polymers.

Next, changes of ^1^H MAS NMR spectra of the 50% plasticized PHA/50% PCL blend with the addition of compatibilizers are shown in Fig. 3B. Based on the signal shapes and intensities of 50% plasticized PHA/50% PCL blend without additives [Fig. 3B(a)], the compatibilizers scarcely changed the ^1^H signal shape except that the addition of PERHEXA25B-40 slightly changed the amount of overlapping ^1^H signals [Fig. 3B(c)]. Therefore, changing the PCL ratio and the addition of both crosslinkers and graft polymers scarcely changed the molecular mobility involved in *T*_2_ relaxation.

### ^13^C MAS NMR spectra of plasticized PHA/PCL blends

We have already evaluated the crystallinity and mobility of the plasticized PHA copolymers and their constituent polymers using a combination of ^13^C CP-MAS NMR and ^13^C PST-MAS NMR methods.^24^ The former CP-MAS method enhances signals of rigid portions utilizing magnetization transfer from ^1^H nuclei to ^13^C nuclei. The latter PST-MAS method enhances signals of flexible portions near hydrogen atoms using the Nuclear Overhauser Effect (NOE).^23^ Here, molecular mobility changes due to the blending ratio and compatibilizers were examined by comparison of CP-MAS and PST-MAS signal intensities for each substituent.

**Fig. 4:**
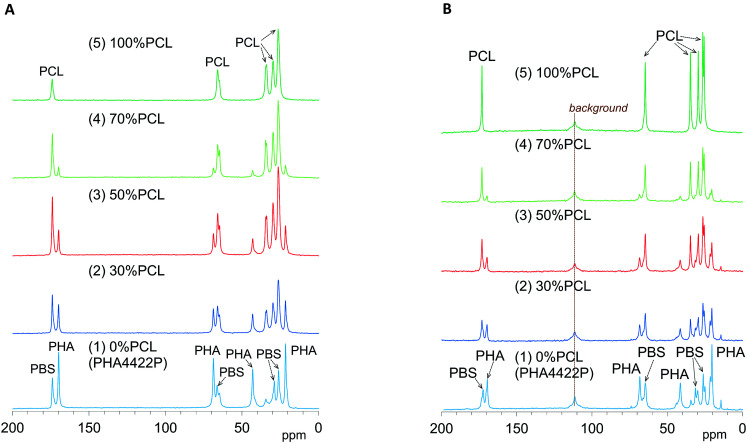
^13^C MAS NMR spectral changes of plasticized PHA/PCL blends with PCL ratio. (A) ^13^C CP-MAS NMR. (B) ^13^C PST-MAS NMR.

Fig. 4A shows changes of ^13^C CP-MAS NMR spectra of plasticized PHA/PCL blends with varying PCL content and Fig. 4B shows their ^13^C PST-MAS NMR spectra. As described in our previous report, the plasticized PHA (PHA4422P) gave sharp signals for the PHA and PBS constituents, which were readily assigned in the ^13^C CP-MAS NMR spectrum [Fig. 4A(1)]. Since PHA was more rigid than PBS, the C=O and CH signals of the PHA moiety in the ^13^C PST-MAS NMR spectrum [Fig. 4B(1)] showed a lower ratio of plasticized PHA to PCL than did the ^13^C CP-MAS NMR spectrum. However, the CH_3_ signal of the PHA moiety in the ^13^C PST-MAS NMR spectrum had a high intensity in a similar manner to the PBS moiety because the CH_3_ group has a high molecular mobility even in rigid polymer like PHA. For flexible polymer such as PCL, the ^13^C CP-MAS NMR spectrum [Fig. 4A(5)] showed one C=O and four CH_2_ signals, of which the highest field signal was assigned as an overlapped signal of two CH_2_ groups. In the ^13^C PST-MAS NMR spectrum [Fig. 4B(5)], the overlapped signal could be separated and the intensities of signals increased with the result that PCL showed six sharp signals.

In the ^13^C CP-MAS NMR spectra of the plasticized PHA/PCL blends [Fig. 4A(2)–(4)], the PCL signals except that at 34 ppm (α-CH_2_) overlapped with the signals derived from PBS in the plasticized PHA. In the ^13^C PST-MAS NMR spectra of the plasticized PHA/PCL blends [Fig. 4B(2)–(4)], the PCL signals except for α-CH_2_ still overlapped with the PBS signals. Therefore, the molecular mobility and dynamics of the plasticized PHA/PCL blend is best discussed using the PHA signals and the α-CH_2_ signal of PCL. At the same time, the molecular mobility and dynamics of PBS in the plasticized PHA/PCL blend can be discussed by comparison of the α-CH_2_ of PCL and other overlapped PCL/PBS signals. Although the PHA signals in both the ^13^C CP- and PST-MAS NMR spectra decreased with increasing the PCL content of the plasticized PHA/PCL, the ratio of PST-MAS signal to CP-MAS signal remained almost constant. In contrast, the ^13^C PST-MAS NMR signal of the α-CH_2_ of homopolymer (5) had a higher intensity than the ^13^C CP-MAS NMR signal. This ratio of PST-MAS signal to CP-MAS signal was reduced by blending with the plasticized PHA. Since PST-MAS emphasizes signals of flexible portions, this result indicates that the blending with the plasticized PHA suppressed the molecular mobility of PCL.

**Fig. 5:**
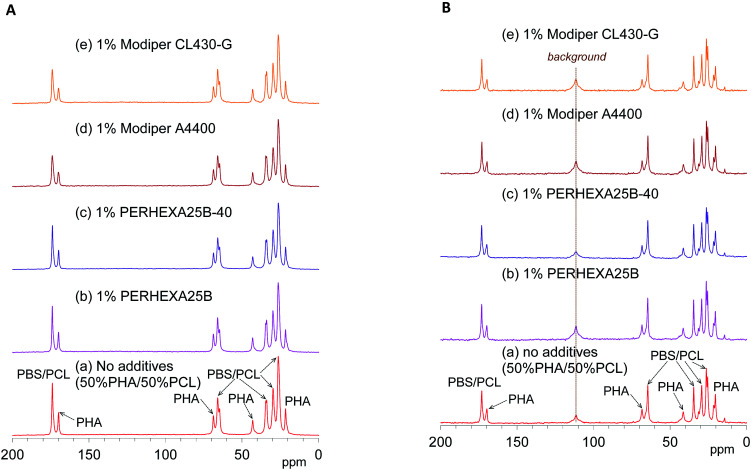
^13^C MAS NMR spectral changes of 50% plasticized PHA/50% PCL blend with compatibilizers. (A) ^13^C CP-MAS NMR. (B) ^13^C PST-MAS NMR.

The effects of the compatibilizers on molecular mobility were also evaluated by the combination of ^13^C CP-MAS NMR and ^13^C PST-MAS NMR methods. Fig. 5A shows changes of the ^13^C CP-MAS NMR spectra of the 50% plasticized PHA/50% PCL blend with the compatibilizers and Fig. 5B their ^13^C PST-MAS NMR spectra. The addition of graft polymers (Modiper A4400, Modiper CL430-G) decreased the C=O signal intensity of PBS/PCL in the ^13^C CP-MAS NMR spectra [Fig. 5A(d) and (e)], compared with the ^13^C PST-MAS NMR spectra [Fig. 5B(d) and (e)], even though the crosslinkers (PERHEXA 25B, PERHEXA 25B-40) exhibited smaller differences between CP-MAS [Fig. 5A(b) and (c)] and PST-MAS [Fig. 5B(b) and (c)]. The combination of the two ^13^C MAS NMR methods showed that the compatibilizers scarcely changed the molecular mobility involved in NOE except that the graft polymers slightly enhanced the molecular mobility of the C=O groups of PBS/PCL.

### Spin-lattice relaxation in the laboratory frame of plasticized PHA/PCL blends

As shown in our preceding study, with increasing the measurement temperature, the *T*_1_H values of pre-plasticized PHA increased while the *T*_1_H values of PBS homopolymer decreased. In the plasticized PHA (PHA/PBS copolymer), however, the *T*_1_H values of the PHA moiety were shortened by the copolymerization with PBS at a higher temperature, because the *T*_1_H relaxation of PHA moiety was enhanced *via* PBS, which had shorter *T*_1_H value than the PHA moiety.^24^ On the other hand, the *T*_1_H value of PCL homopolymer decreased with increasing temperature as did that of the PBS homopolymer. As shown in Fig. S2 (ESI†), the PCL (5 : 100% PCL) showed shorter *T*_1_H values than the constituent polymers in the plasticized PHA (1 : 0% PCL).

**Fig. 6:**
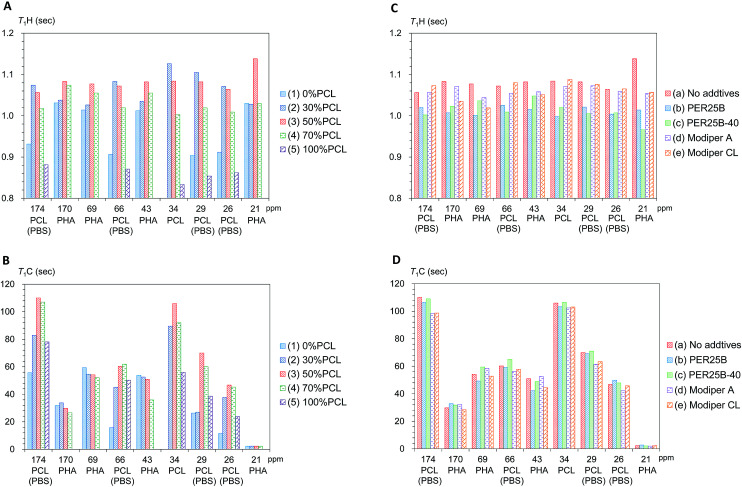
Changes of *T*_1_H and *T*_1_C values of plasticized PHA/PCL blends. (A) *T*_1_H changes with PCL ratio. (B) *T*_1_C changes with PCL ratio. (C) *T*_1_H changes with compatibilizers. (D) *T*_1_C changes with compatibilizers.

First we examined the effects of the blending with PCL on the *T*_1_H value change for each substituent in the plasticized PHA/PCL (Fig. 6A). Even though PCL had shorter *T*_1_H values than the plasticized PHA, the *T*_1_H values of the PHA moiety in the plasticized PHA/PCL blends were increased by the addition of PCL. The *T*_1_H values of PHA moiety attained a maximum value at 50% PCL addition (3). According to the results of the micro-focus X-ray CT, the increase in the *T*_1_H values of the PHA moiety were caused by the change to the sea-island morphology. That is, the *T*_1_H relaxation of PHA, accelerated by the addition of PBS, was suppressed by the sea-island morphology. A similar situation applies to the PCL moiety (α-CH_2_) in the plasticized PHA/PCL blends, where *T*_1_H values increased with an increasing fraction of PHA (decreased PCL) because of the inhomogeneous morphology.

The *T*_1_C value can provide information on molecular motions for each substituent because its value is less affected by ^1^H spin diffusion than the *T*_1_H value. Actually, the *T*_1_C change for each substituent as a function of the PCL content showed a different trend from the *T*_1_H change (Fig. 6B). The *T*_1_C values of the PHA moiety decreased with increasing PCL content: they attained minimum *T*_1_C values at 70% PCL addition (4). In contrast, the α-CH_2_ of PCL moiety obviously had a maximum *T*_1_C value at 50% PCL addition (3). As did the *T*_1_C values of pre-plasticized PHA (CH_2_),^24^ the *T*_1_C values of PCL homopolymer (5) decreased with increasing temperature (Fig. S3, ESI†). The *T*_1_C value provides information about rapid molecular motions (MHz order) of polymer constituents in plasticized PHA/PCL blends. Since the *T*_1_C values of all substituents except CH_3_ group decreased with increasing temperature, the molecular motions connected with these *T*_1_C values are associated with the long correlation time region (*τ*_c_*ω*_0_ ≫ 1).^29^ Therefore, for the components in plasticized PHA/PCL blends, the increase of *T*_1_C equates to a decrease of molecular mobility of a MHz order, which matches with the Larmor frequency of ^13^C nuclei. The *T*_1_C changes associated with blending with 50% PCL show that the sea-island morphology in plasticized PHA/PCL blends considerably decreased the molecular mobility of PCL. In summary, the ^13^C MAS NMR changes due to blending with PCL revealed that the *T*_1_C decrease of PHA in plasticized PHA/PCL blends was caused by the increase of the molecular mobility due to the interaction between PHA and PCL.

Next, changes of *T*_1_H values with the compatibilizers were examined for each polymer component in the 50% plasticized PHA/50% PCL blend (Fig. 6C). Interestingly, the long *T*_1_H values produced by the blending with PCL were shortened again by the addition of crosslinkers (PERHEXA 25B, PERHEXA 25B-40). The *T*_1_H values of the 50% plasticized PHA/50% PCL blend containing the crosslinkers had similar *T*_1_H values to those of the plasticized PHA without PCL. Meanwhile, the graft polymers (Modiper A4400, Modiper CL430-G) produced almost unchanged *T*_1_H values of the PCL and PBS moieties but slightly decreased *T*_1_H values of the PHA moiety. The morphology observed by micro-focus X-ray CT with addition of compatibilizers is closely related to the change of the *T*_1_H value. The crosslinkers produced a homogenous morphology that enhanced the *T*_1_H relaxation of the polymers constituents while the graft polymers maintained the sea-island structure, resulting only in a small change in the *T*_1_H relaxation of PHA.

The effect of compatibilizer on the *T*_1_C values showed marked changes from the *T*_1_H values, as shown in Fig. 6D. The crosslinkers (PERHEXA 25B, PERHEXA 25B-40) scarcely changed the *T*_1_C values of the 50% plasticized PHA/50% PCL blend, resulting in only small decreases for PHA (CH and CH_2_ groups) using PERHEXA 25B. The graft polymers (Modiper A4400, Modiper CL430-G) caused *T*_1_C decreases of the overlapped PCL/PBS signals in the 50% plasticized PHA/50% PCL blend. Although both crosslinker and graft polymer changed mechanical properties, the mechanism by which these changes were manifested differed between crosslinker and graft polymer. That is, the crosslinkers changed morphology uniformly while producing little change in the molecular mobility of PHA, while the graft polymers increased the molecular mobility of PBS in the blend without changing the morphology.

### Spin-lattice relaxation in the rotational frame of plasticized PHA/PCL blends

Due to the cross-polarization produced by the CP-MAS pulse sequence, the ^13^C signal increased with the magnetization transfer from ^1^H nuclei to ^13^C nuclei and, at the same time, the ^13^C signal decreased with the ^1^H spin-lattice relaxation in the rotational frame (*T*_1ρ_H relaxation). Therefore, the changes of *T*_1ρ_H relaxation can be monitored by the observation of the arrayed ^13^C CP-MAS NMR spectra at various contact times.

**Fig. 7:**
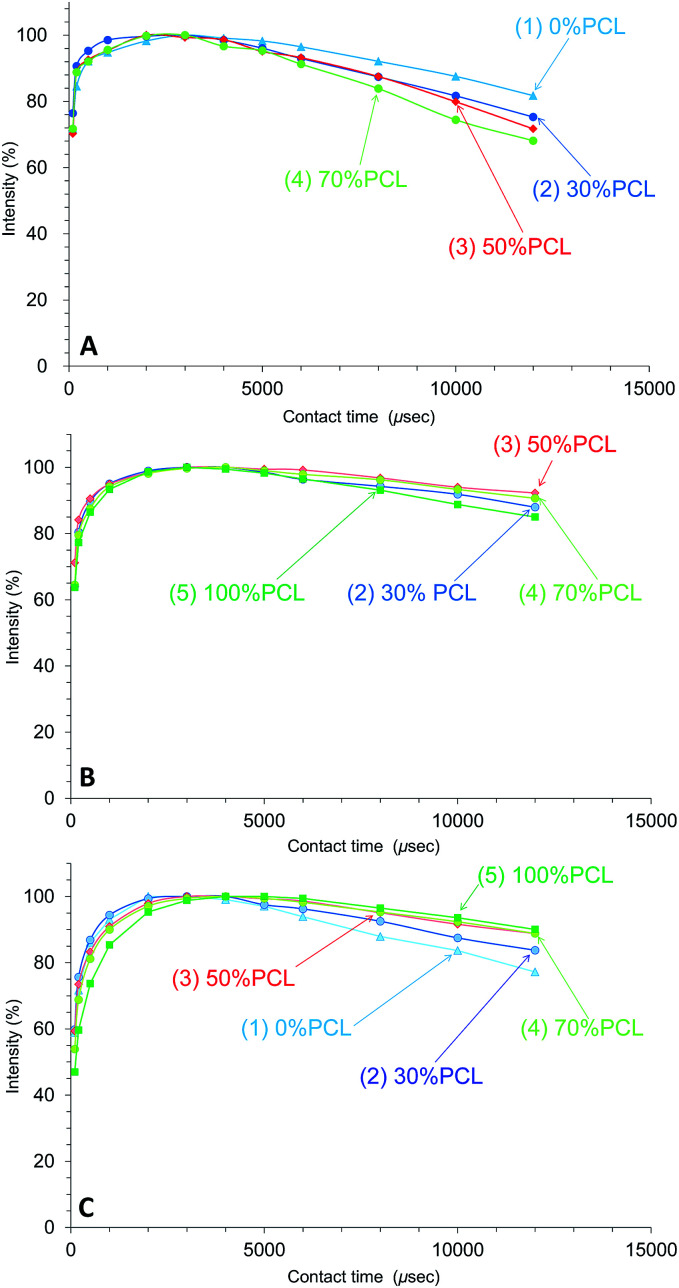
Peak intensity changes of plasticized PHA/PCL blends *versus* contact time in ^13^C CP-MAS NMR. (A) PHA, CH_2_ (43 ppm). (B) PCL, CH_2_ (34 ppm). (C) PBS-PCL, CH_2_ (25 ppm).

Fig. 7 shows the CH_2_ signal intensity curves *versus* contact time for the plasticized PHA/PCL blends for various PCL contents. For the CH_2_ group, the *T*_1ρ_H relaxation of the PHA moiety was more enhanced with the addition of PCL, with the result that the ^1^H spin-lattice relaxation time in the rotational frame (*T*_1ρ_H) of PHA-CH_2_ decreased with increasing PCL content (Fig. 7A). Although the *T*_1ρ_H relaxation of PCL-α-CH_2_ was suppressed in the 50% PHA/50% PCL blend, it proceeded more rapidly with increasing PCL content over 50% (Fig. 7B). Furthermore, the *T*_1ρ_H relaxation of the overlapping CH_2_ features of PBS/PCL was suppressed to various extents depending on the PCL content; that is, the *T*_1ρ_H of PBS decreased with increasing PCL content (decreasing PHA with PHA content) (Fig. 7C). Therefore, the addition of PCL into the plasticized PHA (the PHA/PBS copolymer) suppressed the *T*_1ρ_H relaxation of PBS (long *T*_1ρ_H value), which compensated for the enhanced *T*_1ρ_H relaxation of PHA (short *T*_1ρ_H value) resulting from the immiscible morphology as well as the *T*_1_H relaxation.

**Fig. 8:**
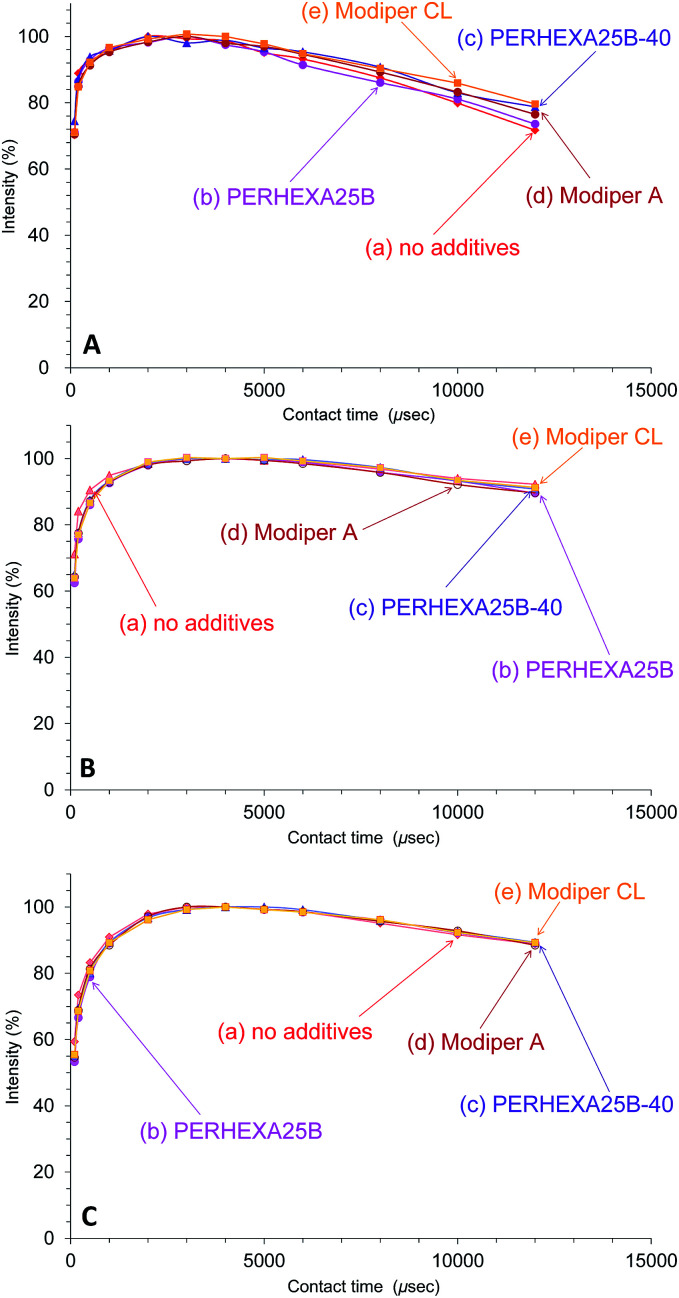
Peak intensity changes of 50% plasticized PHA/50% PCL blends *versus* contact time in ^13^C CP-MAS NMR. (A) PHA, CH_2_ (43 ppm). (B) PCL, CH_2_ (34 ppm). (C) PBS-PCL, CH_2_ (25 ppm).

In contrast to the effect of PCL addition, the *T*_1ρ_H relaxation changed little with various compatibilizers, as shown in Fig. 8. The *T*_1ρ_H relaxation of PHA-CH_2_ was slightly suppressed by the compatibilizers, especially by Modiper CL (Fig. 8A). For CH_2_ groups in both PCL (Fig. 8B) and overlapping signals from PBS/PCL (Fig. 8C), neither crosslinker or graft polymer produced any significant change in the signal decay curve. That is, the compatibilizers slightly lengthened the *T*_1ρ_H value of PHA while they scarcely changed the *T*_1ρ_H values of PCL and PBS. We conclude that *T*_1ρ_H relaxation is concerned with slow molecular mobility (kHz order) and is not related to the tensile properties but could be an indicator of interactions within constituent polymers of the PHA-based blend.

### Multi-scale instrumental analyses of PHA/PCL blends for the effects of blend ratio and additives

**Fig. 9:**
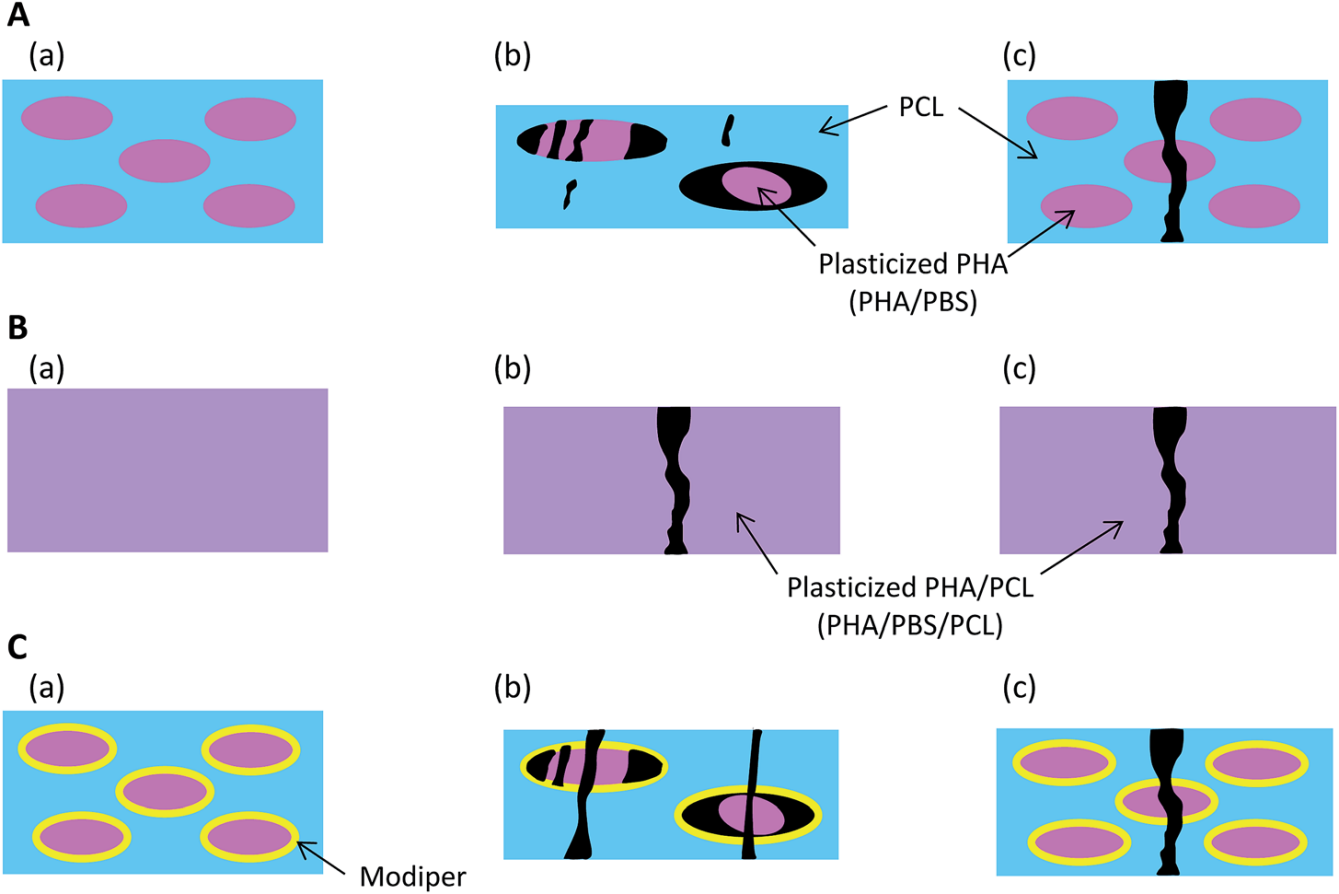
Nanostructural changes of 50% plasticized PHA/50% PCL blend after tensile tests. (A) No additives, (a) before tensile test, (b) after quasi-static test, (c) after dynamic test. (B) Crosslinker, (a) before tensile test, (b) after quasi-static test, (c) after dynamic test. (C) Graft copolymer, (a) before tensile test, (b) after quasi-static test, (c) after dynamic test.

Based on the micro-focus X-ray CT observation (Fig. 2), Fig. 9 presents schematics of the nanostructural changes of the 50% plasticized PHA/50% PCL blend with/without the compatibilizers after the tensile test. Without compatibilizers [Fig. 9(A)], the blend did not interact with PCL and formed the sea-island morphology seen before tensile tests (a). Although PBS in the blend had a similar chemical structure to PCL, the interaction between the plasticized PHA and PCL was weak in the sea-island morphology. In the quasi-static test (b), the plasticized PHA was difficult to extend, even though PCL readily extended. Because of the weak interaction between the plasticized PHA and PCL, the plasticized PHA in the blend adopted a broken island morphology and exfoliated to form the interface with PCL. In the dynamic tensile test (c), since PCL was also difficult to extend, the 50% plasticized PHA/50% PCL blend was easily broken at a small elongation.

By the addition of a crosslinker into the 50% plasticized PHA/50% PCL blend [Fig. 9(B)], the plasticized PHA became compatible with PCL (a). Both plasticized PHA and PLC integrated to produce a homogenous morphology over tens of micron. In the quasi-static test (b), the unification of the plasticized PHA and PCL restrained stretching of the PCL. The plasticized PHA/PLC blend was easily broken at a small elongation because the quasi-static test was dominated by the rigidity of the PHA moiety. Furthermore, the elongation at break in the dynamic tensile test (c) was improved by the addition of cross-linker because the dynamic test was dominated by the flexibility of the PCL moiety.

On the addition of the graft polymer [Fig. 9(C)], although the 50% plasticized PHA/50% PCL blend still adopted the sea-island morphology, the interaction between the plasticized PHA and PCL became strengthened (a), as confirmed by the relaxation time analyses. However, the unifying effect of the graft polymer on the plasticized PHA/PLC blend was weaker than with the crosslinker. Therefore, both plasticized PHA and PCL were broken with a larger elongation at break in the quasi-static test (b). Since the unification of the plasticized PHA/PLC blend mobilized the plasticized PHA and PCL together, the elongation at break was also increased by the addition of graft polymer in the dynamic test (c).

## Conclusions

With the aim of extending application of multi-scale instrumental analyses to biodegradable polymers, plasticized polyhydroxyalkanoates (PHA), containing 65% PHA, 30% poly(butylene succinate) (PBS), and 5% crosslinker, were examined we respect to blending with poly(ε-caprolactone) (PCL) as well as the addition of compatibilizers, such as crosslinkers and graft polymers. Blending PHA with more than 50% PCL increased the tensile strength and elongation at break in the quasi-static tensile test, although the tensile properties were independent of PCL content in the impact tensile test. The crosslinkers markedly lowered the elongation at break in the quasi-static tensile test, while the graft polymers only produced a slight decrease. Both crosslinkers and graft polymers increased the tensile strength and elongation at break in the impact tensile test. Micro-focus X-ray CT observations revealed a sea-island morphology in the 50% plasticized PHA/50% PCL blend. Although the morphology was slightly changed by the addition of graft polymers having acrylonitrile–styrene copolymers as a side-chain, it became homogenous over distances of more than ten microns with the addition of peroxy crosslinkers. The combination of ^13^C CP- and PST-MAS NMR spectra showed that the molecular mobility of PCL was suppressed by blending with the plasticized PHA while the compatibilizers left the molecular motions almost unchanged, with the exception of the C=O groups of PBS/PCL. The ^1^H spin-lattice relaxation time (*T*_1_H) values of the plasticized PHA/PCL blend increased in the sea-island morphology at 50% PCL addition. The lengthened *T*_1_H value of the 50% plasticized PHA/50% PCL blend was shortened again by the crosslinkers, which also changed the sea-island morphology into a homogenous mixture. The *T*_1_H value of blend was shortened to a smaller degree by the graft polymers, which also produced less change in the sea-island morphology. Blending with the plasticized PHA increased the ^13^C spin-lattice relaxation time (*T*_1_C) value of PCL, indicating that the sea-island morphology decreased the molecular mobility of the added PCL. Meanwhile, the *T*_1_C values of the PHA moiety decreased with increasing PCL content to indicate that the added PCL amplified the molecular mobility of PHA. Concerning the effects of compatibilizer on the *T*_1_C values, the graft polymers noticeably decreased the *T*_1_C value of the PBS constituent. The ^1^H spin-lattice relaxation in the rotational frame (*T*_1ρ_H relaxation) of PHA was enhanced by suppression of the *T*_1ρ_H relaxation of PBS because of the immiscible morphology, while the *T*_1ρ_H relaxation was little changed by the addition of compatibilizers. The molecular mobility of the polymers constituents in the plasticized PHA was changed by blending with PCL and the change of molecular mobility caused the increase of tensile strength and elongation at break in the quasi-static tensile test. In contrast, the molecular mobility of the blend was not changed by the crosslinker but was by the graft polymer, while the morphology was not changed by the graft polymer but was by the crosslinker. For these reasons, a different trend for the tensile properties in quasi-static and impact tests was observed when using either crosslinkers or graft polymers. In conclusion, to improve the tensile properties of biodegradable polymers, it is necessary not only to add a component having superior tensile properties but also to match the interaction and molecular mobility of the new component with the original polymer. Considering the tensile properties from the interactions between the constituent polymers, the graft polymer was suitable for polymer blends having different molecular mobility, such as plasticized PHA/PCL blends. In contrast, the crosslinker would be more suitable for polymer blends having similar molecular mobility to each other. NMR is a good indicator of success as the *T*_1_H and *T*_1ρ_H values, which are the indexes involved in the tensile properties, decrease with an increasing interaction while the *T*_1_C value decrease with increasing the molecular mobility. Our present study showed that the multi-scale instrumental analyses gave information about molecular mobility and morphology concerned with the tensile properties of synthetic polymers. Studies of materials characteristics are currently in progress for not only synthetic polymers, but also for natural polymers, using multi-scale instrumental analyses.

## Conflicts of interest

There are no conflicts to declare.

## Supplementary Material

RA-009-C8RA10045D-s001
