# Supplementary material for: Multi-scale instrumental analyses of plasticized polyhydroxyalkanoates (PHA) blended with polycaprolactone (PCL) and the effects of crosslinkers and graft copolymers
Source: RSC Adv. 2019 Jan 11;9(3):1551–61. doi: 10.1039/c8ra10045d (PMC9059665; doi:10.1039/c8ra10045d)
Supplement: RA-009-C8RA10045D-s001 [file RA-009-C8RA10045D-s001.pdf]

Electronic Supplementary Material (ESI) for RSC Advances.
This journal is © The Royal Society of Chemistry 2019

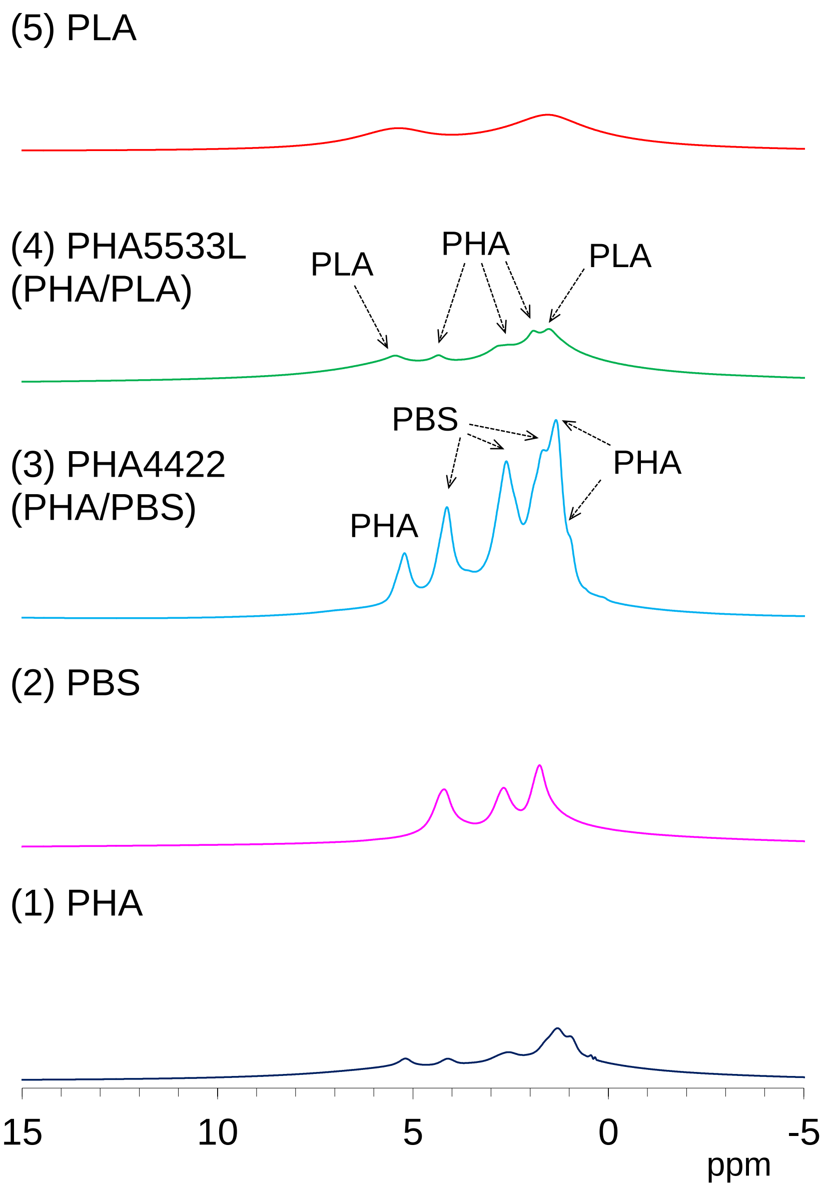

Fig. S1. ^{1}H MAS NMR spectra of plasticized PHA and constituent polymers.

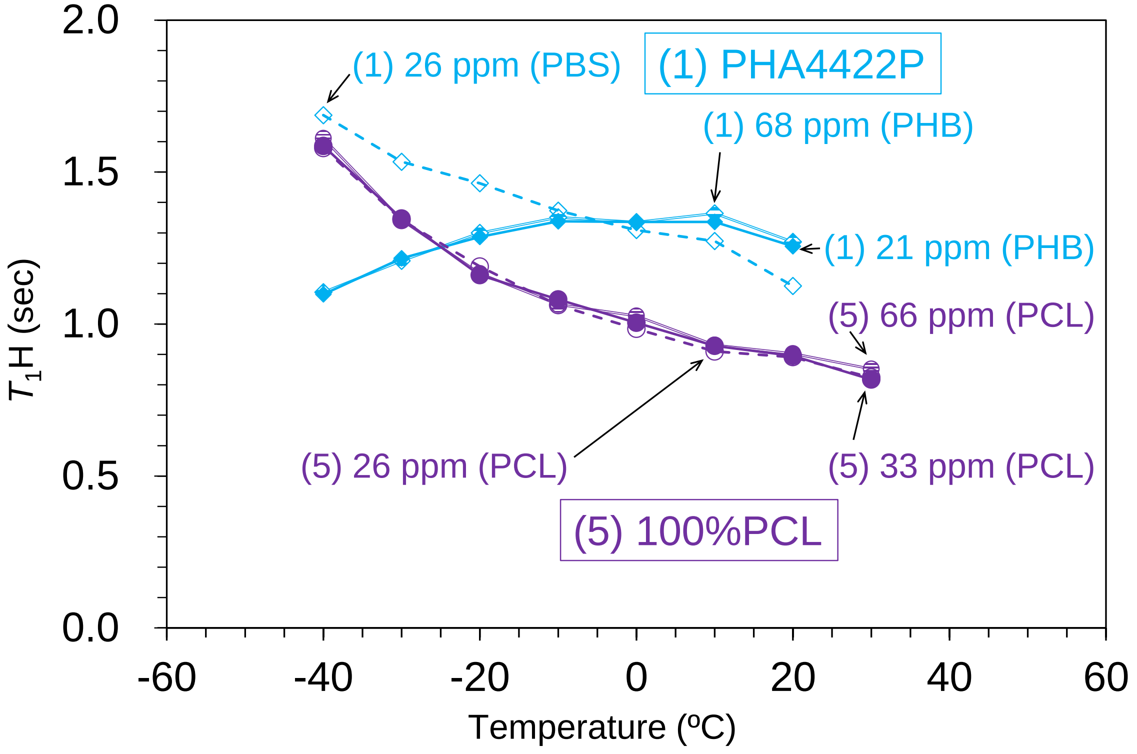

Fig. S2. T_1H changes of plasticized PHA (1) and PCL (5) with rising temperature.

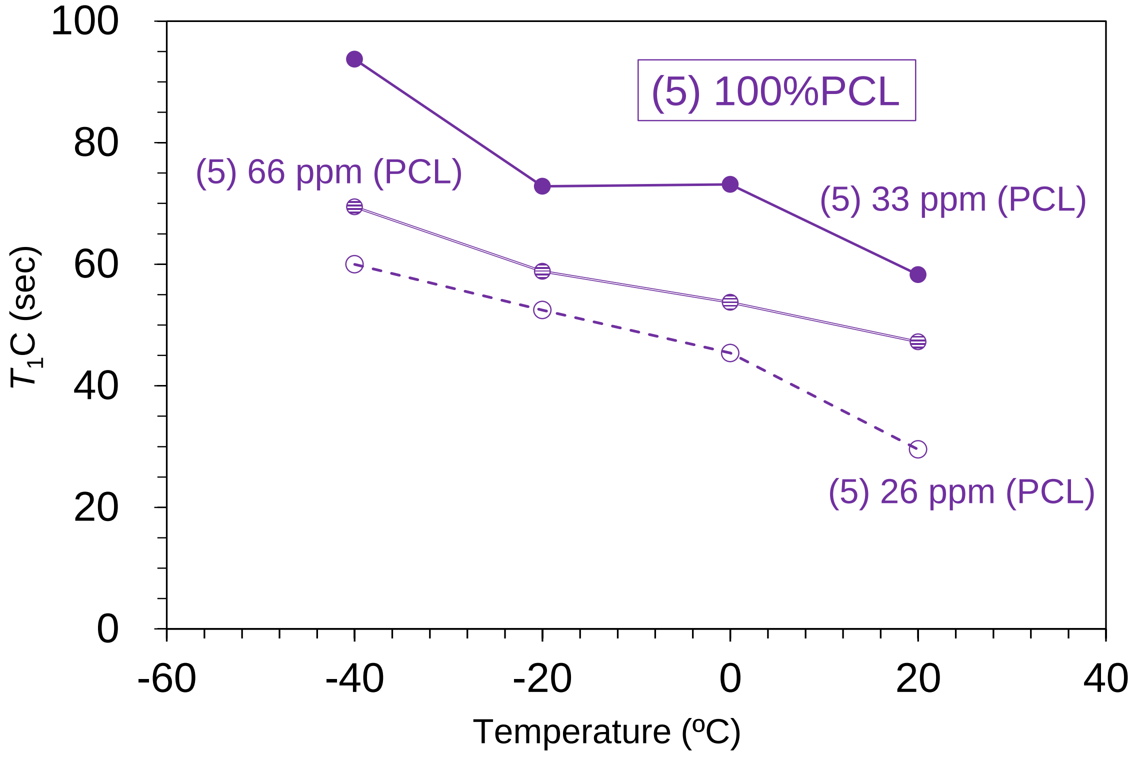

Fig. S3. T_1C changes of PCL (5) with rising temperature.
